# Ionizing radiation reduces ADAM10 expression in brain microvascular endothelial cells undergoing stress-induced senescence

**DOI:** 10.18632/aging.101225

**Published:** 2017-04-17

**Authors:** Lucinda S. McRobb, Matthew J. McKay, Jennifer R. Gamble, Michael Grace, Vaughan Moutrie, Estevam D. Santos, Vivienne S. Lee, Zhenjun Zhao, Mark P. Molloy, Marcus A. Stoodley

**Affiliations:** ^1^ Department of Clinical Medicine, Faculty of Medicine and Health Sciences, Macquarie University, Sydney, New South Wales, 2109, Australia; ^2^ Australian Proteome Analysis Facility, Department of Chemistry and Biomolecular Sciences, Macquarie University, Sydney, New South Wales, 2109, Australia; ^3^ Vascular Biology Program, Centenary Institute, University of Sydney, Sydney, New South Wales, 2042, Australia; ^4^ Genesis Cancer Care, Macquarie University Hospital, Sydney, New South Wales, 2109, Australia

**Keywords:** endothelial cells, senescence, ionizing radiation, ADAM10, biotinylation

## Abstract

Cellular senescence is associated with aging and is considered a potential contributor to age-associated neurodegenerative disease. Exposure to ionizing radiation increases the risk of developing premature neurovascular degeneration and dementia but also induces premature senescence. As cells of the cerebrovascular endothelium are particularly susceptible to radiation and play an important role in brain homeostasis, we investigated radiation-induced senescence in brain microvascular endothelial cells (EC). Using biotinylation to label surface proteins, streptavidin enrichment and proteomic analysis, we analyzed the surface proteome of stress-induced senescent EC in culture. An array of both recognized and novel senescence-associated proteins were identified. Most notably, we identified and validated the novel radiation-stimulated down-regulation of the protease, a disintegrin and metalloprotease 10 (ADAM10). ADAM10 is an important modulator of amyloid beta protein production, accumulation of which is central to the pathologies of Alzheimer's disease and cerebral amyloid angiopathy. Concurrently, we identified and validated increased surface expression of ADAM10 proteolytic targets with roles in neural proliferation and survival, inflammation and immune activation (L1CAM, NEO1, NEST, TLR2, DDX58). ADAM10 may be a key molecule linking radiation, senescence and endothelial dysfunction with increased risk of premature neurodegenerative diseases normally associated with aging.

## INTRODUCTION

Exposure to ionizing radiation (IR) is linked with an increased risk of developing cardiovascular and neurovascular diseases normally associated with aging [1-5]. In the brain, radiation has been associated with development of cerebral amyloid angiopathy (CAA), where toxic amyloid beta (Aβ) plaques accumulate in perivascular regions causing hemorrhage and stroke [5]. Radiation is also a risk factor for early dementia, however links to an increased risk of Alzheimer's disease (AD) lack solid epidemiological evidence, despite the fact that AD is characterized by Aβ plaque formation in the brain parenchyma and that CAA occurs in more than 80% of AD patients [6-8].

While neurons are considered relatively resistant to radiation-induced cell death, endothelial cells (ECs) are particularly susceptible [9, 10]. There is increasing recognition that damage or dysfunction of the neurovascular unit in the microvasculature may progressively affect cerebral blood flow and brain clearance mechanisms, leading to accumulation of toxic metabolites, neuronal death and progressive cognitive decline [11, 12]. Studies suggest that initial progression of AD may be partially driven by early neurovascular degeneration [13].

Cellular senescence is associated with aging in various brain cell types, including neurons and ECs, and has been associated with neurodegenerative disease [14]. Senescence is characterized by the induction of irreversible cell growth arrest, however cells remain metabolically and transcriptionally active. Replicative senescence describes cell arrest after finite population doublings and is typically associated with reductions in telomere length in cultured cells and highly proliferative tissue [15]. Premature stress-induced senescence is more common in non-proliferating cells and is caused primarily by oxidative stress and DNA damage [16]. The term “geroconversion” has also been coined to describe the cellular transition in aging from quiescent to “typically senescent”, which renders cells gerogenic (able to cause organismal aging) and pathogenic (able to cause disease) [17]. Senescent cells down-regulate the production of proliferative proteins but increase production of pro-inflammatory and pro-thrombotic molecules, expressing a senescence-associated secretory phenotype (SASP) that promotes leukocyte infiltration and stimulates the innate immune response [18]. Radiation has been shown to stimulate a stress-induced premature senescence-like phenotype in ECs both *in vitro* and *in vivo* [19-21]. In this study, we aimed to examine radiation-stimulated changes in ECs entering senescence *in vitro* to increase our understanding of the molecular mechanisms that may link radiation, senescence and age-associated neurodegenerative disorders.

Proteins at the surface of cerebral endothelial cells communicate with both the blood and the underlying brain and hence play a critical role in signalling and transport across the blood-brain barrier. Biotin-labelling is a well-established approach to tag and subsequently enrich membrane and surface-accessible proteins from cell or tissue extracts [22-24]. Here we employ *in vitro* biotin labelling, mass spectrometry and proteomic analysis to examine changes in the surface proteome of irradiated brain microvascular ECs entering senescence. Examining the surface proteome may identify proteins subject to post-translational alterations affecting subcellular localization or protein trafficking, changes missed by traditional whole-cell proteomic or microarray studies. In addition, identification of unique surface markers may potentially allow development of novel therapeutic approaches to target removal or attenuation of inflammatory senescent cells through vascular targeting [25-27]. Here we document for the first time the radiation-stimulated changes in the surface proteome of brain ECs in culture undergoing stressinduced senescence and discuss the potential significance to the early stages of neurodegeneration.

## RESULTS

### Radiation inhibits proliferation, induces hypertrophy and cell death in brain microvascular endothelial cells

**Figure 1:**
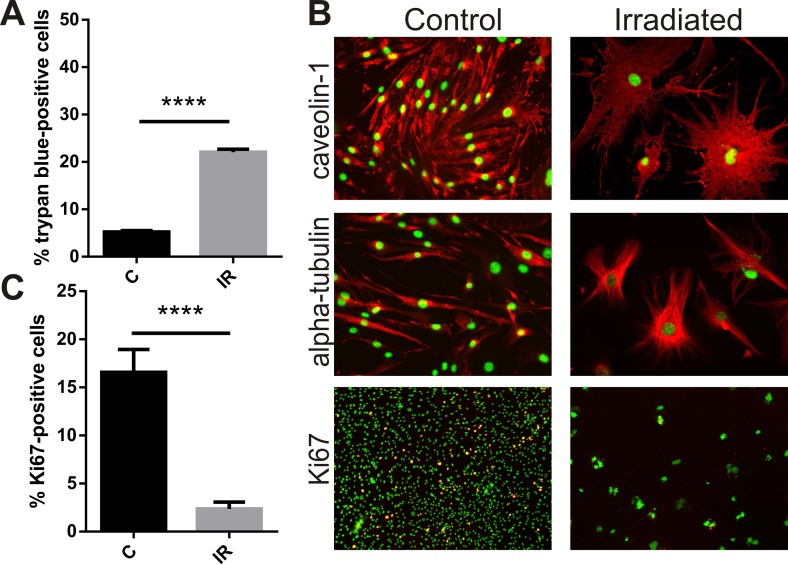
Radiation inhibits proliferation, induces cell death and hypertrophy in brain endothelial cells Mouse bEnd.3 cells were delivered a dose of 20 Gy ionizing radiation by linear accelerator. (**A**) Floating and adherent cells were collected at day 6 and cell viability/death measured by trypan blue staining and counting in a Neubauer chamber. Data show trypan blue positive cells as a percentage of total cells. Mean of 3 independent experiments ± SEM. (**B**) Representative images of non-irradiated (control) and irradiated bEnd.3 cells stained after permeabilization for caveolin-1 or α-tubulin showing cell hypertrophy and cytoskeletal rearrangement at day 6 (red, 200× magnification), or proliferation marker Ki67 (lower panels, red, 100× magnification). Nuclei were counterstained with DAPI (green). Co-localization (yellow). (**C**) The proportion of Ki67 positive cells was determined using Image J. Data represent mean ± SEM calculated in 5 fields of view (100×) from 2 independent experiments. Student's *t*-test, ****P<0.0001.

Exposure of bEnd.3 cells to a single 20 Gy dose of radiation increased cell death as measured by the trypan blue viability assay. Six days after irradiation or sham treatment the proportion of dead cells in the irradiated group was 4-fold higher than in the non-irradiated cell population (P<0.0001) (Fig. 1A). For bEnd.3 cells that did not undergo apoptosis and die, adherent cells remaining after day 6 post-IR demonstrated a clear change in cellular morphology (Fig. 1B). Cells became flattened and hypertrophic with significant changes in cell shape and cytoskeletal structure (caveolin and alpha-tubulin staining, Fig. 1B). Cells positive for the proliferation marker Ki67 were significantly reduced in populations of radiation-stimulated cells (P< 0.0001) (Fig. 1B, C).

### Radiation induces cellular senescence

**Figure 2:**
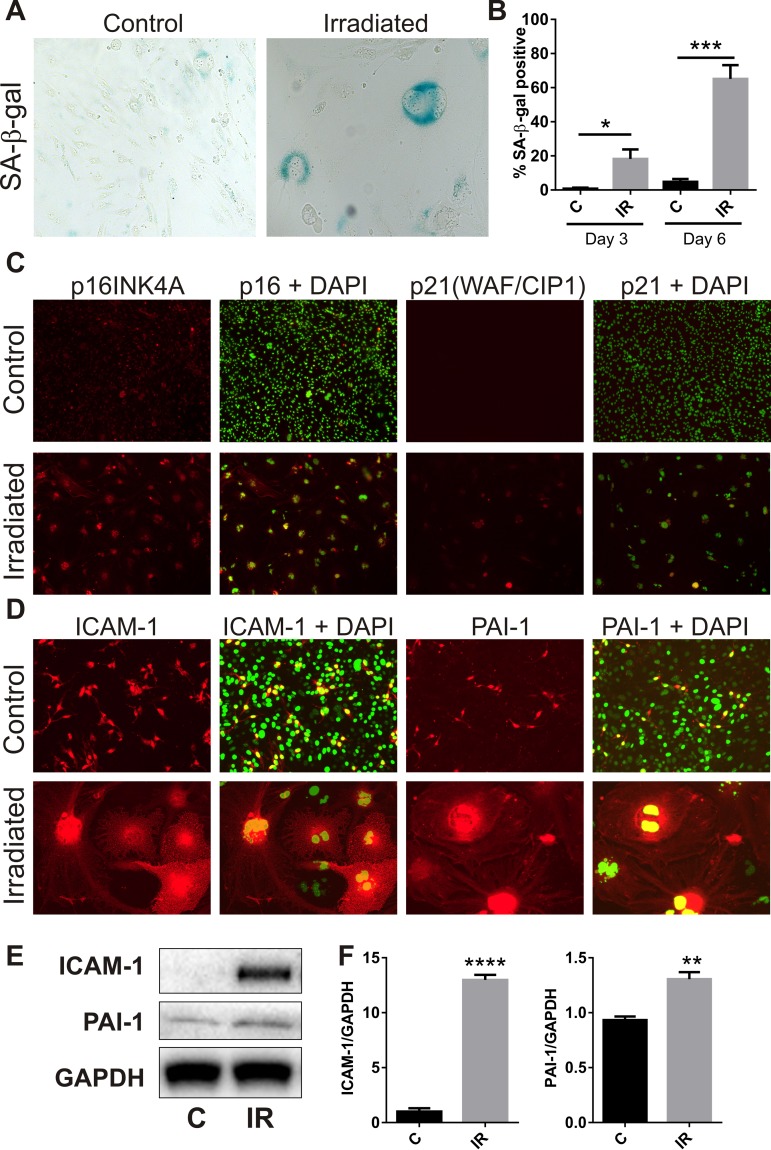
Radiation induces senescence-associated markers (**A**) Representative bright field images of non-irradiated (control) and irradiated bEnd.3 cells stained for lysosomal SA-β-gal activity (perinuclear blue staining) at day 6 post-IR or sham (100× magnification). (**B**) The proportion of SA-β-gal-positive cells was quantitated at day 3 and day 6 in 3–4 independent experiments. (**C**) Representative immunofluorescent images of nuclear accumulation of CDK inhibitors, p21 and p16 (red), in irradiated cells after 6 days (red, 100× magnification). (**D**) Representative immunofluorescent images of ICAM-1 and PAI-1 staining in control and irradiated cells after 6 days (red, 200× magnification). Cells were counterstained with cell surface marker wheat germ agglutinin conjugated to AF488 (blue). Cell nuclei were stained with DAPI in all merged images (green). (**E**) ICAM-1 and PAI-1 expression were determined in control and irradiated cells by western blotting and quantitated after normalization to GAPDH using Image J (Figure F; n=4 independent experiments). All data are shown as mean ± SEM. Student's *t*-test *P<0.05, *** P<0.01, ***P<0.001, ****p<0.0001. C, control; IR, irradiated.

The majority of bEnd.3 cells remaining adherent 6 days post-irradiation demonstrated increased activity of the lysosomal enzyme, SA-β-Gal (Fig. 2A), a marker of cell senescence [28]. The proportion of SA-β-Gal positive cells reached 18 ± 6% at day 3 (P<0.05) and 65 ± 8% by day 6 (P<0.001) (Fig. 2B). Immunocytochemistry showed increased polyploidy, the presence of lobed nuclei and nuclear expression of the senescence-associated cyclin-dependent kinase (CDK) inhibitors, p21 (WIF/CAP) and p16 (INK4A) (Fig. 2C), as well as increased expression of the senescence markers intercellular adhesion molecule 1 (ICAM-1) and plasminogen activator inhibitor 1(PAI-1) (Fig. 2D) [29-31]. Western analysis of whole cell lysates confirmed up-regulation of ICAM-1 (P<0.0001) and PAI-1 (P<0.01) at the protein level (Fig. 2E, F).

### Radiation alters autophagic flux

**Figure 3:**
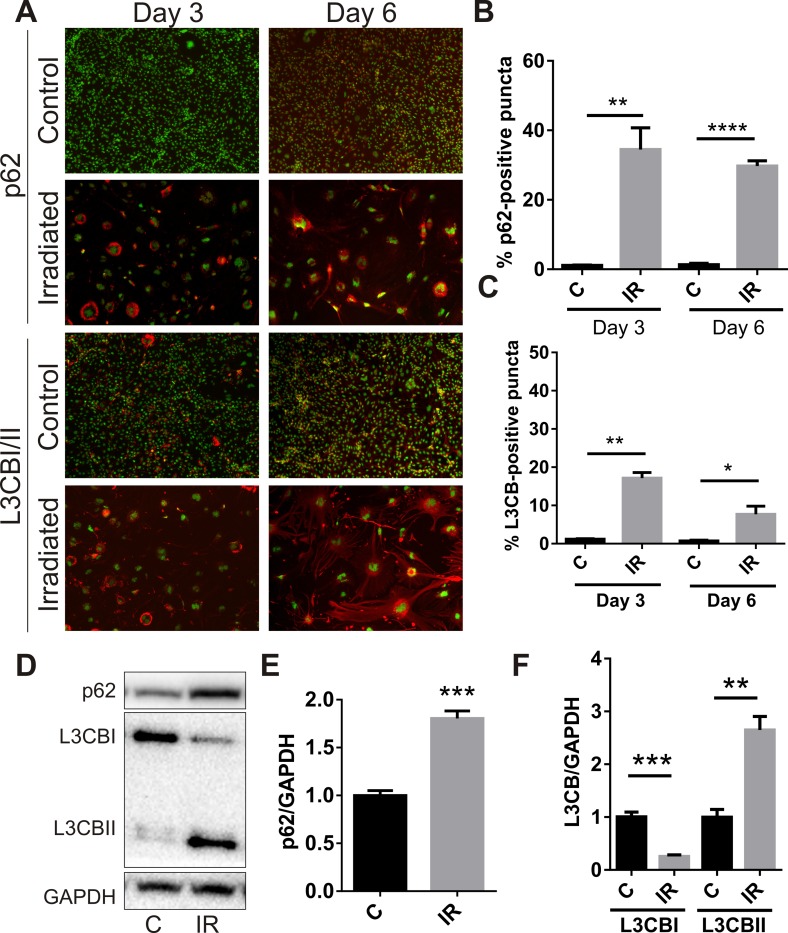
Radiation stimulates accumulation of autophagy-associated markers in brain endothelial cells bEnd.3 cells were delivered a dose of 20 Gy ionizing radiation and monitored for 3–6 days. (**A**) Representative immunofluorescent images showing increased perinuclear p62 or L3CB-positive puncta accumulating in permeabilized cells at day 3 and day 6 post-IR or sham (red, 100× magnification). Cells were counterstained with DAPI (green). The percentage of cells positive for p62 (**B**) or L3CBI/II puncta (**C**) were quantitated using Image J (n=3 independent experiments; positive cells were counted in n=8 fields of view). (**D**) Representative western blots of p62, L3CBI and II autophagosomal markers in whole cell lysates of control and irradiated cells after 6 days. (**E**) and (**F**) Bands were quantitated after normalization to GAPDH using Image J (n=4 independent experiments). All data are shown as mean ± SEM. Student's *t*-test, *P<0.05, *** P<0.01, ***P<0.001, ****p<0.0001. C, control; IR, irradiated.

A recent study associated senescence with simultaneous or prior alterations in autophagy or autophagic flux [32], therefore accumulation of the autophagosomal proteins, p62 and microtubule-associated protein L3CBI/II, was examined. Immunocytochemistry demonstrated peri-nuclear accumulation of p62 in adherent cells at day 3 (34 ± 6%, P<0.01) and day 6 (29 ± 2%, P<0.0001) (Fig. 3A, B). Perinuclear accumulation of L3CB was also observed (17 ± 2% at day 3, P<0.01; 8 ± 2% at day 6, P<0.05) (Fig. 3A, C). In opposition to that observed for SA-β-Gal, the number of cells positive for perinuclear L3CB and p62 puncta appeared to decrease over time. Western analysis demonstrated increased total protein levels of p62 (Fig. 3D, E) and almost total conversion of L3CBI to the lipidated L3CBII form in irradiated cells by day 6 (Fig. 3D, F), changes consistent with a radiation–induced blockade of autophagic flux [33].

### Proteomic and ingenuity pathway analysis of biotinenriched proteins

Proteomic analysis of streptavidin-biotin enriched fractions from cells 6 days post-IR or sham led to the identification of 647 proteins in total across both sham and irradiated cell extracts, of which 205 proteins were considered differentially regulated at a fold change threshold of 1.5. Of the 73 proteins increased at the cell surface greater than 1.5-fold in response to radiation (Supplementary Table S2), 31 were considered statistically significant (P<0.05). Of the 132 proteins decreased 1.5-fold at the cell surface in response to radiation (Supplementary Table S3), 50 were considered statistically significantly (P<0.05). Selected proteins identified as differentially expressed in the biotin-enriched extracts were chosen for further validation.

Causal network analysis was performed using the IPA platform (Supplementary Table S4). Network associations were cellular movement and cell growth and proliferation. The top upstream regulators included MYC, N-MYC and p53, well-known to regulate the switch between apoptosis and senescence [34]. In addition, inclusion of “sirolimus” (otherwise known as rapamycin) as an upstream regulator is consistent with studies demonstrating that radiation can induce senescence in EC through inhibition of the PI3K/AKT/MTOR (mammalian target of rapamycin) pathway [35]. Western analysis confirmed that radiation caused a chronic reduction in AKT phosphorylation at day 6 (not shown). The top canonical pathways included EIF2 signalling and regulation of eIF4 and p70S6K signalling, both downstream pathways of mTOR, with a role in regulating protein translation, as also observed previously in response to low dose radiation in EC [35].

### Radiation reduces expression of the alpha-secretase and ectodomain sheddase, ADAM10

**Figure 4:**
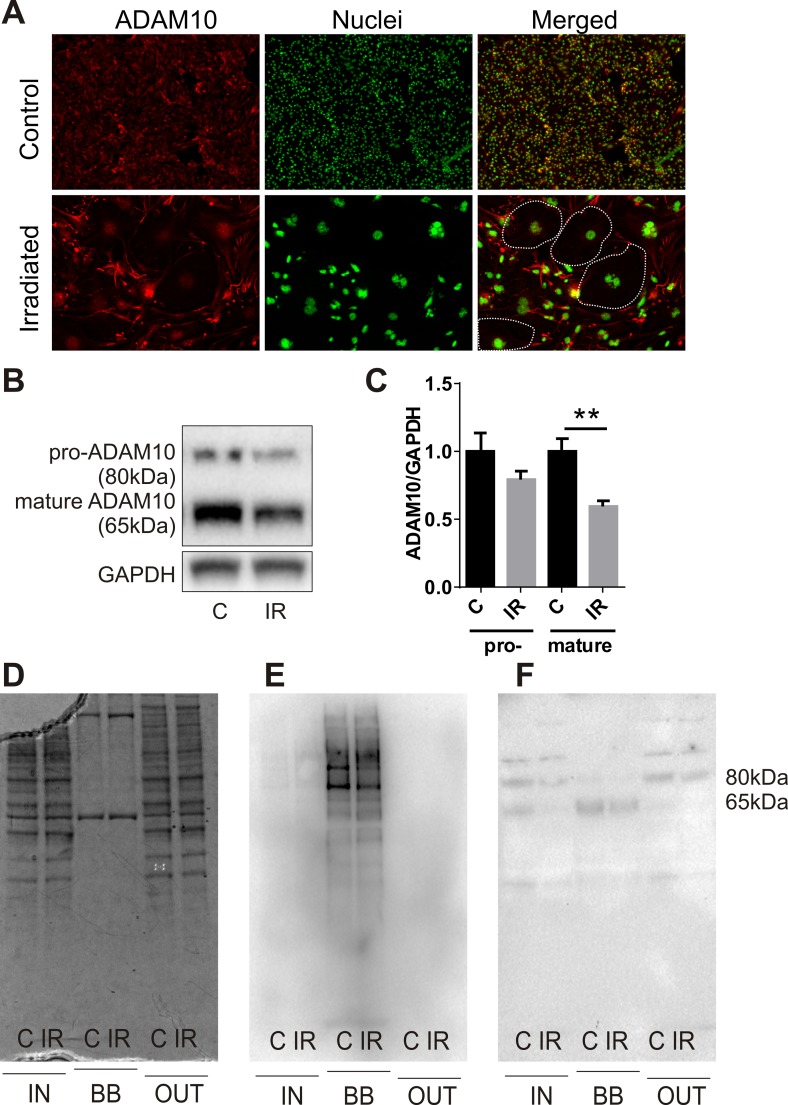
Radiation modulates expression of the alpha-secretase, ADAM10 (**A**) Representative immunofluorescent images of ADAM10 expression 6 days after radiation (20 Gy) or sham treatment; ADAM10 (red), DAPI-stained nuclei (green); 100× magnification, n=2 independent experiments. Dotted lines in merged images indicate the boundary of several large senescent cells with reduced ADAM10 immunostaining. (**B**) Representative western blots of whole cell lysates (15 μg) probed for pro-ADAM10 and mature ADAM10. (**C**) Protein bands (ADAM10) were quantitated after normalization to GAPDH using Image J. Data represent mean ± SEM of 4 independent experiments. Student's *t*-test **P<0.01. (**D**–**F**) Representative images of fractionated extracts (10 μg each lane) from biotin-labelled cells before and after streptavidin enrichment: (**D**) Coomassie-stained SDS-PAGE gel; (**E**) streptavidin-HRP-probed membrane; (**F**) anti-ADAM10-probed membrane. IN, total cell extract prior to streptavidin enrichment; BB, biotin-bound fraction; OUT, non-biotinylated fraction eluted post-streptavidin binding; C, control; IR, irradiated.

Examination of the proteomic data revealed the novel radiation-stimulated down-regulation of ADAM10 (a disintegrin and metalloprotease 10) (2.5-fold, P=0.05; Table S3). ADAM10 is an alpha-secretase and ectodomain sheddase that plays an important role in the post-translational cleavage of multiple proteins both intracellularly and at the cell surface [36]. ADAM10 is the protease responsible for cleavage of the amyloid precursor protein (APP) in the brain to a soluble neuroprotective fragment (sAPPα). APP cleavage by the beta-secretase, BACE-1, precludes formation of toxic amyloid beta (AB) peptides, accumulation of which contributes to the pathophysiology of CAA and AD [36, 37]. Validation of ADAM10 down-regulation by immunocytochemistry demonstrated that high basal expression of this protein in non-irradiated cells was reduced in response to radiation and appeared to associate with the hypertrophic senescent cells (Fig. 4A). Western analysis confirmed that total protein levels of both the pro and mature (active) forms of ADAM10 were reduced in response to radiation (Fig. 4B, C). In addition, western analysis of biotin-tagged cell extracts after streptavidin-enrichment confirmed the surface expression of the mature but not pro-ADAM10 protein and its downregulation in response to radiation (Fig. 4D-F).

### Radiation increases surface localization of ADAM10 target proteins

**Figure 5:**
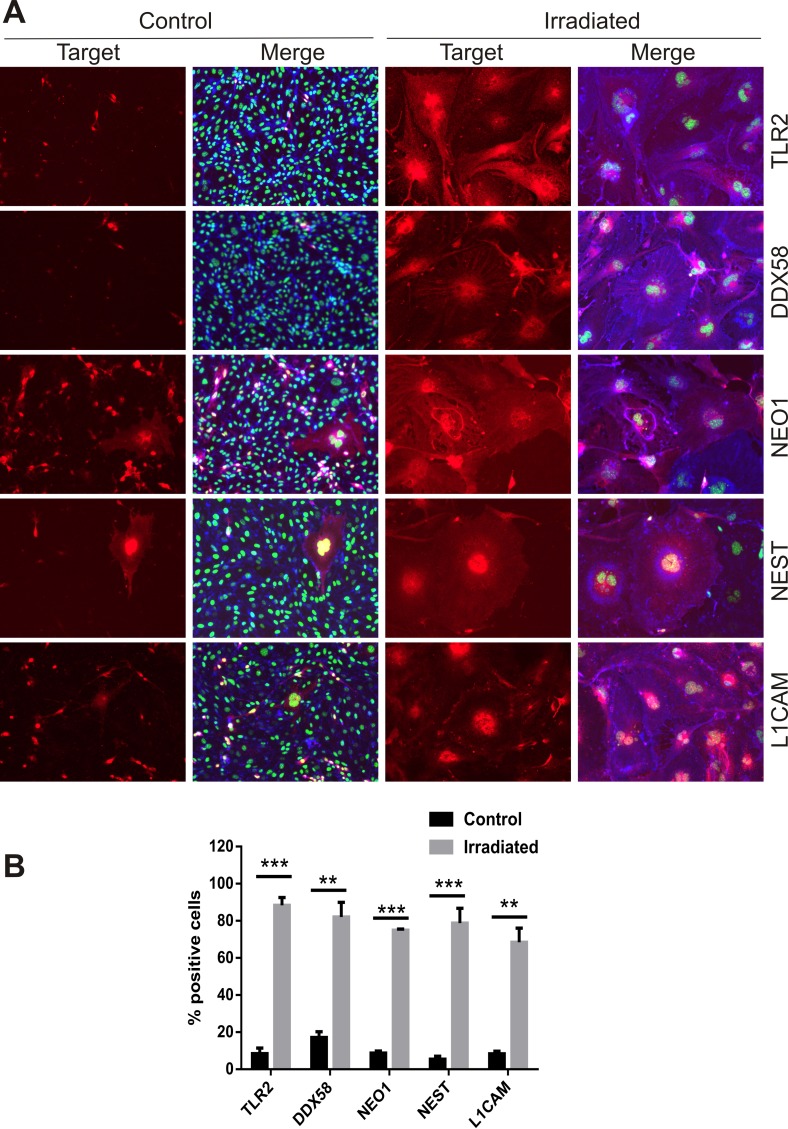
Immunofluorescent localization of ADAM10 target proteins (**A**) Representative immuno-fluorescent images of non-permeabilized cells stained for L1CAM, NEST, NEO1, DDX58 and TLR2 after 6 days post-IR or sham (controls). Cells were co-stained with DAPI (nuclei, green) and wheat germ agglutinin–AF488 (surface marker, blue). All images are shown at 200× magnification. (**B**) Percentage of cells staining positively for each target protein 6 days after IR or sham. Data represent at least 3 independent experiments, positive cells were counted in n=3 fields of view. All data shown as mean ± SEM. Student's *t*-test **P<0.01, ***P<0.001. C, control; IR, irradiated.

Proteins known to be targets of ADAM10 proteolytic activity, either direct or indirect, were identified as concurrently up-regulated at the cell surface in the proteomic datasets. Neural cell adhesion molecule L1 (L1CAM), Nestin (NEST), Neogenin (NEO1), Toll-like receptor 2 (TLR2) and an ATP-dependent RNA helicase, DDX58 (otherwise known as retinoic acid inducible gene 1 or RIG-1) play key roles in neuro-inflammation and innate immune activation [38-40] and were further validated. Immunocytochemical staining of non-permeabilized cells at day 6 post-IR or sham confirmed the increased surface expression of L1CAM, NEST, NEO1, TLR2 and DDX58 in response to radiation (Fig. 5A). The proportion of positively stained cells rose from 5–20% in sham-irradiated cells to 70–90% of the cell population after irradiation (Fig. 5B). In addition, it was noted that hypertrophic, multinucleated senescent-like cells observed sporadically in the sham-irradiated cell populations also demonstrated externalization of these proteins, suggesting surface expression was associated with both replicative and stress-induced senescence (Fig. 5A).

## DISCUSSION

Radiation exposure is associated with an increased risk of age-associated neurodegenerative disease [3-5]. As aging is associated with a progressive increase in cellular senescence, there is a growing interest in both senescence and dysfunctional autophagy as facilitators of neurodegeneration and AD [41, 42]. In this study, we have shown that radiation can stimulate stress-induced senescence in association with blockade of autophagic flux in brain microvascular ECs. For the first time we show that this stress-induced senescence is associated with down-regulation of the alpha-secretase, ADAM10. Further, we have identified concomitant up-regulation of ADAM10 target proteins at the cell surface that may contribute to radiation-stimulated neuroinflammation and immune activation.

In the current study, a single high dose of ionizing radiation induced both apoptosis and cellular senescence in brain microvascular ECs *in vitro* consistent with previous studies that have shown moderate radiation doses can induce senescence in ECs of various origins [43-46]. Radiation also triggered perinuclear accumulation of the autophagosomal proteins, p62 and L3CBII, suggesting blockade of autophagic flux [33], replicating a recent study performed in human umbilical vein endothelial cells [47]. While autophagy and senescence are often regarded as separate survival pathways, autophagy blockade can induce senescence in various cell types [32]. Perinuclear accumulation of p62 and L3CB appeared to peak transiently prior to establishment of a primarily SA-β-Gal-positive population in this study, suggesting a similar temporal association. The radiation induced senescence and autophagy dysfunction was associated with reduced AKT/MTOR kinase signalling in pathway analysis, a finding consistent with recent studies showing: low dose radiation suppresses MTOR and AKT activity in ECs; PI3K/AKT inhibition induces both endothelial senescence and autophagy [35, 48, 49]. Recent studies coined the term “geroconversion” to describe the irreversible step between quiescence and senescence. A key role for MTOR activation in the permanent transitioning from replicative to non-replicative cell was suggested which is also responsible for the hypertrophy associated with a senescence-like phenotype [17, 50]. A mechanism through MTOR activation conflicts with the current findings and those in earlier publications using ECs. This may suggest a transient MTOR activation peak is missed in these studies or that cell-type specific pathways exist depending on the stimulus. Further studies are required to define the signalling pathways controlling radiation–induced senescence in vascular ECs.

Biotin labelling is a well-established method to label surface-accessible proteins for enrichment from complex protein lysates prior to proteomic analysis. Ours is the first study to use this approach to examine surface changes in stress-induced senescent cells. The limited abundance of membrane and surface proteins relative to intracellular proteins often precludes their identification in proteomic analysis which has a limited dynamic range. Biotin-labelling allows potential recognition of proteins that may alter their abundance or subcellular localization by post-translational processes and may go undetected using microarrays or proteomic analysis of whole cell lysates. Identification of novel senescence-associated surface proteins may identify potential markers amenable to vascular targeting to treat or remove senescent cells as a therapeutic approach to treat age-associated disease [25-27]. Our own studies are investigating the use of radiation as a priming agent to induce novel marker expression for vascular targeting in brain arteriovenous malformations, of which radiation-induced senescence markers could be of use [51-53]. Identifying markers at the endothelial surface may have many applications for understanding and subsequently attenuating age-associated disease.

ADAM10 was highly down-regulated by radiation stress and has not been associated previously with senescence, although a recent study demonstrated that ADAM10 can be regulated by autophagy or autophagy impairment in ECs [54]. Alzheimer's disease (AD) is associated with reduced levels of ADAM10 in the human brain while ADAM10 over-expression has been shown to improve cognitive function in mouse models of AD [55]. One important target of ADAM10 in the brain is the transmembrane amyloid precursor protein (APP). ADAM10 competes with the beta-secretase, BACE-1, for APP [55]. Cleavage of APP by ADAM10 stimulates the non-amyloidogenic pathway via formation of soluble APPα fragments, which have neurotrophic and neuroprotective properties. In contrast, BACE-1 cleavage of APP results in the eventual production of toxic Aβ peptides. ADAM10 cleaves the APP protein within the Aβ domain, precluding the formation of Aβ peptides. Aβ accumulation is characteristic of CAA and AD and plays a major role in their pathogenesis [37, 55]. In CAA, Aβ peptides accumulate within the perivascular rather than parenchymal regions of the brain, causing vascular damage and microhemorrhage [37]. Although CAA occurs in more than 80% of AD patients, radiation has not been epidemiologically linked to AD, despite suggested associations [7]. Further, a recent study demonstrated that radiation reduces Aβ peptide burden and improves cognition in an AD mouse model [56]. Although we did not identify the APP protein in our data set as being regulated, hence we did not further validate any surface changes in this study, this does not preclude alteration in its function as a result of ADAM10 changes after radiation.

In addition to non-amyloidogenic APP cleavage, ADAM10 contributes to neuroprotection and immunomodulation through the ectodomain shedding of numerous molecules [57, 58], of which several identified in the proteomic data set were validated. L1CAM and NEO1 are known ADAM10 targets [57, 59]. NEO1 is normally found on the surface of growing nerve cells and plays a role in axon guidance during development [38]. L1CAM acts as a receptor for proinflammatory T-cell binding at the cell surface [40]. Cleavage and release of the L1 fragment promotes neuron proliferation and survival [60]. TLR2 and DDX58 are also ADAM10 targets [61-63]. These proteins are damage-associated molecular pattern (DAMP) receptors that activate the innate immune response when bound by stress-induced ligands not of viral or bacterial origin [39]. Cleavage and release of the soluble TLR2 ectodomain suppresses TLR2 activity by binding and quenching DAMP ligands, an activity thought to reduce the development of autoimmunity through a negative feedback loop [61]. DDX58 is indirectly regulated by ADAM10, through cleavage of the anti-ageing protein, Klotho [62, 63]. Klotho is highly abundant in the brain and considered an important regulator of aging-related inflammation and protection against dysfunction. NEST is a type VI intermediate filament protein characteristic of neuronal stem cells. NEST is not normally present at the cell surface however there is previous evidence for its cell surface translocation in glioma cells [64]. NEST has been suggested to play a role in radio-protection [65]. Both ADAM10 and gamma-secretase activity appear to post-translationally regulate NEST expression and subcellular localization [64, 66]. Overall, the proteins identified in this study and their role in inflammation and innate immune activation reinforce the notion that radiation regulation of ADAM10 may be a key molecular link to neurovascular inflammation and ultimately, premature neurodegeneration reminiscent of aging.

## CONCLUSIONS

There is an increasing interest in understanding both cellular senescence and autophagy and their contribution to neurovascular disease and neurodegeneration associated with aging. Radiation is an important risk factor for age-related diseases such as CAA and dementia, and the radiation-stimulated downregulation of ADAM10 identified here in senescent cells suggests ADAM10 may be a key player in the molecular etiology of radiation risks for CAA, dementia and potentially AD.

## METHODS

### Cell culture and irradiation

Cells from the ATCC-derived murine brain endothelial cell line, bEnd.3, were cultured in Dulbecco's Modified Eagles Medium (4.5 g/L glucose, 3.7 g/L sodium bicarbonate, 4 mM glutamine) (Invitrogen), supplemented with 10% fetal bovine serum, penicillin (100 U/mL) and streptomycin (0.1 mg/mL) and maintained at 37°C in humidified 95% air with 5% carbon dioxide. Cells were used between passages 18-24 and passaged with 0.1% Trypsin/EDTA. Cells were seeded in 6-well plates for protein extraction, 8-well chamber slides (Thermoscientific) for immunocytochemistry or 75 cm^2^ flasks for biotinlabelling at 30% confluence and irradiated with X-rays (20 Gy) generated by a 6 MV linear accelerator (LINAC, Elekta Synergy, Crawley, UK) at Macquarie University Hospital (Sydney, Australia) as previously described [67]. Control cells were treated identically but received no radiation.

### Trypan blue viability assay

Viable-to-dead cell ratios were determined 6 days after irradiation using a Neubauer chamber and trypan blue exclusion. Briefly, both floating and adherent cells were collected, washed and stained with trypan blue for 10 min and 10 μL transferred in duplicate to a Neubauer chamber and the number of live (white) to dead (blue) cells were counted.

### Senescence-associated β-galactosidase activity assay

Senescence-associated β-galactosidase (SA-β-Gal) activity was determined according to the manufacturer's instructions (Abcam, ab65351) [28]. The bEnd.3 cells plated in 8-well chamber slides at a density of 2 × 10^4^ cells/mL were fixed 3 or 6 days post-IR or sham. The percentage of cells with perinuclear blue staining observed in 5 fields-of-view with bright field microscopy (Magnification: 200× for controls; 100× for irradiated cells) was calculated in 3 independent experiments performed in triplicate.

### Immunocytochemistry

Cells grown in 8-well chamber slides were fixed with paraformaldehyde (2%, 5 min) with (0.3% Triton-X100, 15 min) or without permeabilization (no detergents), where specified. Sections were blocked in 5% donkey serum and 1% bovine serum albumin (BSA) prior to overnight staining with primary antibodies at 4°C. Primary antibodies are listed in Supplementary Table S1. Proteins were visualized with species-specific AlexaFluor647-conjugated secondary antibodies (Life Technologies). Cells were co-stained with wheat germ agglutinin (WGA) conjugated to AlexaFluor488 (Life Technologies) to visualize surface proteins and nuclei were counterstained with 4′,6-diamidino-2-phenylindole dihydrochloride (DAPI, 5 μg/mL). Controls incubated with equimolar rabbit IgG (Santa Cruz Biotechnologies) or mouse IgG (BD Biosciences) showed no reactivity. All control sections incubated without primary antibodies demonstrated negative staining. Digital images were captured under fixed parameters using a Zeiss microscope with AxioCam HRc camera and Zen 2012 software (Carl Zeiss Microscopy).

### *In vitro* biotinylation and protein extraction

At day 6 after irradiation or sham, cells in T75 flasks were prepared for biotin labelling, using a modification of published methods [23, 24, 68]. Briefly, cells were washed four times with cold 10 mL phosphate-buffered saline (PBS, pH 7.4) to remove all medium and secreted proteins. EZ-link Sulfo-NHS-LC Biotin (Thermoscientific) was dissolved in PBS (150 μM) and 5 mL added per flask and incubated at room temperature with gentle rocking for 5 min. The biotin was quenched with 1 mM Tris-HCl (pH 7.5) in PBS for 5 min. Cells were then washed four times in 10 mL PBS (5 min each wash). Cells were lysed (2% NP40, 0.2% SDS, 1× protease inhibitor mix (GE Healthcare), 10 mM EDTA in PBS) and collected by scraping with a rubber policeman. Lysis was continued for 30 min on ice prior to sonication (40% power, 3 × 15s). The supernatant was clarified by centrifugation at 12000 g for 15 min at 4°C to pellet insoluble debris. An aliquot was then taken to determine protein concentration by BCA assay (Pierce Biotechnology Inc.) before streptavidin enrichment.

### Streptavidin enrichment and on-resin trypsinization

Enrichment on streptavidin-agarose beads was based on published methods [23, 24]. Protein samples were thawed on ice while 600 μL Streptavidin-Sepharose HP slurry (GE Healthcare) was washed three times with 500 μL buffer A (1% NP40, 0.1% SDS in PBS). The slurry was centrifuged at 2000 g for 1 min between washes. Protein extracts were resuspended to a concentration of 1 mg/mL and 1 mL added to the washed resin and mixed by rotation at room temperature to allow binding. After 2 h, the slurry was centrifuged and the supernatant discarded (non-biotinylated fraction). The biotin-bound resin was washed three times with 500 μL buffer A, then twice with buffer B (0.1 % NP40 substitute, 1 M NaCl in PBS). For mass spectrometry analysis, the resin was washed 8 times with 50 mM ammonium bicarbonate (pH 8) containing 0.5% (w/v) sodium deoxycholate, then resuspended in 300 μL of buffer and adjusted to 5 mM dithiothretol (Bio-Rad) in 100 mM ammonium bicarbonate and reduced at 70°C for 60 min. Samples were alkylated with iodoacetamide (Bio-Rad) at a final concentration of 15 mM (in 100 mM ammonium bicarbonate) at room temperature for 1 h. Finally, bound proteins were digested with the addition of 2 μg of trypsin (Promega) and incubation of the beads at 37°C overnight under constant agitation. The supernatant was recovered following centrifugation of samples at 10000 g for 10 min. Sodium deoxycholate was removed by centrifugation (10000 g for 10 min, 3 times) following acidification of samples to a final concentration of 1% formic acid. The supernatant was removed and peptides were concentrated using a SpeediVac and resuspended in 2% acetonitrile (0.1% formic acid) prior to LC/MS analysis.

A separate set of biotinylated extracts were prepared for western analysis. Preparation was identical to that performed for mass spectrometry analysis however the final ammonium bicarbonate washes were replaced with one wash of 50 mM Tris-HCl (pH 7.5) prior to extraction of bound proteins by incubation in a solution of 3 mM biotin, 2% SDS and 8M urea for 15 min at room temperature and 15 min at 95°C.

### LC/MS/MS and data analysis

Samples were analyzed using data-dependent and SWATH LC/MS/MS procedures described previously, with modifications [53, 69]. LC/MS/MS was performed using a TripleTOF 6600 mass spectrometer (AB SCIEX) equipped with a NanoLC™ 400 liquid chromatography system (Eksigent) and cHiPLC unit (Eksigent). Reverse phase separations were conducted using a 200 μm × 0.5 mm nano cHiPLC trap column (ChromXP™ C18-CL 3 μm 120 Å; Eksigent) at a flow rate of 5 μL/min for 5 min (2%ACN, 0.1% FA), and a 150 mm × 200 μm nano cHiPLC column (ChromXP™ C18-CL 3 μm 120 Å) using a linear gradient from 5% to 40% (90% ACN, 0.1% FA) at a flow rate of 600 nL/min over 60 minutes. Peptides were subjected to positive ion nanoflow analysis using an ion spray voltage, heater interface temperature, curtain gas flow and nebulizing gas flow of 2.5 kV, 150°C, 25 and 16 L/min, respective-ly. For data-dependent acquisition experiments, a “top 20” approach utilized a full MS survey scan (350–1250 amu, 250 ms) followed by 20 MS/MS product ion scans (100–1500 amu, 100 ms each). Product ion scans were collected for ions with a 2+ to 4+ charge-state and an ion intensity threshold of 150 counts per second (cps). Spectral libraries were generated by searching product ion data against all mouse entries in the UniProt database (release April 2014, 20266 entries) using the Paragon algorithm in ProteinPilot™ software (V5.0, AB Sciex). An Unused Score of 2.0 (99% confidence) was used as a cut-off, resulting in a protein FDR of <1%. Database searches were conducted with carbamido-methyl modifications of cysteine residues in the thorough ID mode and excluded biological modifications. For data independent SWATH acquisition experiments, a variable windows approach (350–1250 amu) with 100 windows was used.

ProteinPilot results and data-independent SWATH experiments were imported into PeakView™ software 2.0 (AB Sciex) and analyzed with the SWATH MicroApp 2.0. Peak extraction was performed for a maximum of 100 high confidence peptides (99%) per protein and six transitions per peptide, with a tolerance of 75 ppm using a peptide extract threshold FDR of 1%. Protein level quantitative comparisons were assessed by summing ion peak area values for the transitions of each peptide per protein and normalizing the total protein area. Fold changes for each protein were assessed by comparing the natural log of the normalized mean protein areas for each protein and back transforming the natural logs. A two sample *t*-test (0.05) was used to determine the significance of the protein fold change. Proteins with a P value of 0.05 and fold change of < 0.7 or > 1.5 were considered significant.

Causal network analysis of signalling networks in proteomic data was performed using INGENUITY Pathway Analysis (IPA) (INGENUITY System, www.INGENUITY.com). The full set of proteins (642) was used as input, with observations used for the analysis being average fold changes of the irradiated/control conditions. Default Ingenuity parameters were selected; the reference set considered was the default genomics background. The top five identified pathways, networks, tox lists and upstream regulators were tabulated.

### Western blotting

Total proteins from whole cell lysates were extracted from irradiated (20 Gy) and control bEnd.3 cells after 6 days using radioimmunoprecipitation (RIPA) lysis buffer (50 mM Tris–HCl, pH 7.5, 150 mM NaCl, 0.5% deoxycholate, 0.1% sodium dodecyl sulfate (SDS), 1% NP40 substitute, 5 mM EDTA) with protease inhibitor mix (GE healthcare). After lysis, the material was sonicated for 2 min at 30 s intervals and centrifuged at 12000 g for 10 min at 4°C. Protein concentrations were determined using the BCA protein assay (Pierce Biotechnology Inc.) using bovine serum albumin (BSA) as a standard. Equal amounts of whole cell protein extracts (15 μg) were resolved by SDS-PAGE, transferred to a PVDF membrane using the iblot transfer system (ThermoFisher Scientific) and probed with primary antibodies (Supplementary Table S1) and species-specific secondary antibodies conjugated to horseradish peroxidase (HRP). Bands were detected using enhanced chemiluminescence. GAPDH was used as a loading control. NIH Image J open-source software (http://imagej.nih.gov/ij/) was used to quantitate protein bands on blots in 4 independent experiments. Results are expressed as mean ± SEM. Two group comparisons were performed using the unpaired, two-tailed Student's *t*-test.

For biotin-tagged protein extracts, 10 μg each of total extract (IN), biotin-bound extract (BB) and nonbiotinylated, unbound extract (OUT) were resolved by SDS-PAGE as described. Biotin was detected on blots using HRP-conjugated streptavidin (Abcam).

## SUPPLEMENTARY MATERIAL TABLES



## Abbreviations

Ab: amyloid beta
AD: Alzheimer's disease
ADAM10: a disintegrin and metalloproteinase-domain containing protein 10
APP: amyloid precursor protein
BACE-1: beta-site amyloid precursor protein cleaving enzyme 1
BSA: bovine serum albumin
CAA: cerebral amyloid angiopathy
DAMP: damage-associated molecular pattern
DDX58: dead box protein 58/RIG-1-like receptor 1
EC: endothelial cell
FDR: false discovery rate
Gy: Gray
IR: ionizing radiation
L1CAM: neural cell adhesion molecule 1
L3CB: microtubule-associated protein 1 light chain 3 beta, LC/MS, liquid chromatography-mass spectrometry
LINAC: linear accelerator
NEO1: neogenin 1
NEST: nestin
SA-b-gal: senescence-associated beta-galactosidase
SASP: senescence-associated secretory phenotype
SWATH: sequential window acquisition of all theoretical mass spectra
TLR2: toll-like receptor 2

## References

[1] Darby SC, McGale P, Taylor CW, Peto R (2005). Long-term mortality from heart disease and lung cancer after radiotherapy for early breast cancer: prospective cohort study of about 300,000 women in US SEER cancer registries. Lancet Oncol.

[2] Shimizu Y, Kodama K, Nishi N, Kasagi F, Suyama A, Soda M, Grant EJ, Sugiyama H, Sakata R, Moriwaki H, Hayashi M, Konda M, Shore RE (2010). Radiation exposure and circulatory disease risk: hiroshima and Nagasaki atomic bomb survivor data, 1950-2003. BMJ.

[3] Asai A, Matsutani M, Kohno T, Nakamura O, Tanaka H, Fujimaki T, Funada N, Matsuda T, Nagata K, Takakura K (1989). Subacute brain atrophy after radiation therapy for malignant brain tumor. Cancer.

[4] Lowe XR, Bhattacharya S, Marchetti F, Wyrobek AJ (2009). Early brain response to low-dose radiation exposure involves molecular networks and pathways associated with cognitive functions, advanced aging and Alzheimer's disease. Radiat Res.

[5] Sugihara S, Ogawa A, Nakazato Y, Yamaguchi H (1995). Cerebral beta amyloid deposition in patients with malignant neoplasms: its prevalence with aging and effects of radiation therapy on vascular amyloid. Acta Neuropathol.

[6] Jellinger KA (2002). Alzheimer disease and cerebrovascular pathology: an update. J Neural Transm (Vienna).

[7] Begum N, Wang B, Mori M, Vares G (2012). Does ionizing radiation influence Alzheimer's disease risk?. J Radiat Res (Tokyo).

[8] Ghiso J, Frangione B (2002). Amyloidosis and Alzheimer's disease. Adv Drug Deliv Rev.

[9] Dimitrievich GS, Fischer-Dzoga K, Griem ML (1984). Radiosensitivity of vascular tissue. I. Differential radiosensitivity of capillaries: a quantitative in vivo study. Radiat Res.

[10] Belka C, Budach W, Kortmann RD, Bamberg M (2001). Radiation induced CNS toxicity—molecular and cellular mechanisms. Br J Cancer.

[11] Ghiso J, Fossati S, Rostagno A (2014). Amyloidosis associated with cerebral amyloid angiopathy: cell signaling pathways elicited in cerebral endothelial cells. J Alzheimers Dis.

[12] Weller RO, Subash M, Preston SD, Mazanti I, Carare RO (2008). Perivascular drainage of amyloid-beta peptides from the brain and its failure in cerebral amyloid angiopathy and Alzheimer's disease. Brain Pathol.

[13] Hayashi S, Sato N, Yamamoto A, Ikegame Y, Nakashima S, Ogihara T, Morishita R (2009). Alzheimer disease-associated peptide, amyloid beta40, inhibits vascular regeneration with induction of endothelial autophagy. Arterioscler Thromb Vasc Biol.

[14] Chinta SJ, Woods G, Rane A, Demaria M, Campisi J, Andersen JK (2015). Cellular senescence and the aging brain. Exp Gerontol.

[15] Greider CW (1998). Telomeres and senescence: the history, the experiment, the future. Curr Biol.

[16] Toussaint O, Medrano EE, von Zglinicki T (2000). Cellular and molecular mechanisms of stress-induced premature senescence (SIPS) of human diploid fibroblasts and melanocytes. Exp Gerontol.

[17] Blagosklonny MV (2014). Geroconversion: irreversible step to cellular senescence. Cell Cycle.

[18] Lasry A, Ben-Neriah Y (2015). Senescence-associated inflammatory responses: aging and cancer perspectives. Trends Immunol.

[19] Seol MA, Jung U, Eom HS, Kim SH, Park HR, Jo SK (2012). Prolonged expression of senescence markers in mice exposed to gamma-irradiation. J Vet Sci.

[20] Azimzadeh O, Sievert W, Sarioglu H, Merl-Pham J, Yentrapalli R, Bakshi MV, Janik D, Ueffing M, Atkinson MJ, Multhoff G, Tapio S (2015). Integrative proteomics and targeted transcriptomics analyses in cardiac endothelial cells unravel mechanisms of long-term radiation-induced vascular dysfunction. J Proteome Res.

[21] Le ON, Rodier F, Fontaine F, Coppe JP, Campisi J, DeGregori J, Laverdière C, Kokta V, Haddad E, Beauséjour CM (2010). Ionizing radiation-induced long-term expression of senescence markers in mice is independent of p53 and immune status. Aging Cell.

[22] Scheurer SB, Rybak JN, Roesli C, Brunisholz RA, Potthast F, Schlapbach R, Neri D, Elia G (2005). Identification and relative quantification of membrane proteins by surface biotinylation and two-dimensional peptide mapping. Proteomics.

[23] Rybak JN, Ettorre A, Kaissling B, Giavazzi R, Neri D, Elia G (2005). In vivo protein biotinylation for identification of organ-specific antigens accessible from the vasculature. Nat Methods.

[24] Roesli C, Neri D, Rybak JN (2006). In vivo protein biotinylation and sample preparation for the proteomic identification of organ- and disease-specific antigens accessible from the vasculature. Nat Protoc.

[25] Minamino T, Miyauchi H, Yoshida T, Tateno K, Komuro I (2004). The role of vascular cell senescence in atherosclerosis: antisenescence as a novel therapeutic strategy for vascular aging. Curr Vasc Pharmacol.

[26] Velarde MC, Demaria M, Campisi J (2013). Senescent cells and their secretory phenotype as targets for cancer therapy. Interdiscip Top Gerontol.

[27] Tchkonia T, Zhu Y, van Deursen J, Campisi J, Kirkland JL (2013). Cellular senescence and the senescent secretory phenotype: therapeutic opportunities. J Clin Invest.

[28] Dimri GP, Lee X, Basile G, Acosta M, Scott G, Roskelley C, Medrano EE, Linskens M, Rubelj I, Pereira-Smith O (1995). A biomarker that identifies senescent human cells in culture and in aging skin in vivo. Proc Natl Acad Sci USA.

[29] Gorgoulis VG, Pratsinis H, Zacharatos P, Demoliou C, Sigala F, Asimacopoulos PJ, Papavassiliou AG, Kletsas D (2005). p53-dependent ICAM-1 overexpression in senescent human cells identified in atherosclerotic lesions. Lab Invest.

[30] Kortlever RM, Higgins PJ, Bernards R (2006). Plasminogen activator inhibitor-1 is a critical downstream target of p53 in the induction of replicative senescence. Nat Cell Biol.

[31] Matsumura T, Zerrudo Z, Hayflick L (1979). Senescent human diploid cells in culture: survival, DNA synthesis and morphology. J Gerontol.

[32] Kang C, Elledge SJ (2016). How autophagy both activates and inhibits cellular senescence. Autophagy.

[33] Klionsky DJ, Abdelmohsen K, Abe A, Abedin MJ, Abeliovich H, Acevedo Arozena A, Adachi H, Adams CM, Adams PD, Adeli K, Adhihetty PJ, Adler SG, Agam G (2016). Guidelines for the use and interpretation of assays for monitoring autophagy (3rd edition). Autophagy.

[34] Hydbring P, Larsson LG (2010). Cdk2: a key regulator of the senescence control function of Myc. Aging (Albany NY).

[35] Yentrapalli R, Azimzadeh O, Sriharshan A, Malinowsky K, Merl J, Wojcik A, Harms-Ringdahl M, Atkinson MJ, Becker KF, Haghdoost S, Tapio S (2013). The PI3K/Akt/mTOR pathway is implicated in the premature senescence of primary human endothelial cells exposed to chronic radiation. PLoS One.

[36] Saftig P, Lichtenthaler SF (2015). The alpha secretase ADAM10: A metalloprotease with multiple functions in the brain. Prog Neurobiol.

[37] Auriel E, Greenberg SM (2012). The pathophysiology and clinical presentation of cerebral amyloid angiopathy. Curr Atheroscler Rep.

[38] Andrusiak MG, McClellan KA, Dugal-Tessier D, Julian LM, Rodrigues SP, Park DS, Kennedy TE, Slack RS (2011). Rb/E2F regulates expression of neogenin during neuronal migration. Mol Cell Biol.

[39] Rosin DL, Okusa MD (2011). Dangers within: DAMP responses to damage and cell death in kidney disease. J Am Soc Nephrol.

[40] Menzel L, Paterka M, Bittner S, White R, Bobkiewicz W, van Horssen J, Schachner M, Witsch E, Kuhlmann T, Zipp F, Schäfer MK (2016). Down-regulation of neuronal L1 cell adhesion molecule expression alleviates inflammatory neuronal injury. Acta Neuropathol.

[41] Nixon RA, Yang DS (2011). Autophagy failure in Alzheimer's disease--locating the primary defect. Neurobiol Dis.

[42] Nussenzweig SC, Verma S, Finkel T (2015). The role of autophagy in vascular biology. Circ Res.

[43] Igarashi K, Miura M (2008). Inhibition of a radiation-induced senescence-like phenotype: a possible mechanism for potentially lethal damage repair in vascular endothelial cells. Radiat Res.

[44] Oh CW, Bump EA, Kim JS, Janigro D, Mayberg MR (2001). Induction of a senescence-like phenotype in bovine aortic endothelial cells by ionizing radiation. Radiat Res.

[45] Kim KS, Kim JE, Choi KJ, Bae S, Kim DH (2014). Characterization of DNA damage-induced cellular senescence by ionizing radiation in endothelial cells. Int J Radiat Biol.

[46] Ungvari Z, Podlutsky A, Sosnowska D, Tucsek Z, Toth P, Deak F, Gautam T, Csiszar A, Sonntag WE (2013). Ionizing radiation promotes the acquisition of a senescence-associated secretory phenotype and impairs angiogenic capacity in cerebromicrovascular endothelial cells: role of increased DNA damage and decreased DNA repair capacity in microvascular radiosensitivity. J Gerontol A Biol Sci Med Sci.

[47] Kalamida D, Karagounis IV, Giatromanolaki A, Koukourakis MI (2014). Important role of autophagy in endothelial cell response to ionizing radiation. PLoS One.

[48] Breitschopf K, Zeiher AM, Dimmeler S (2001). Pro-atherogenic factors induce telomerase inactivation in endothelial cells through an Akt-dependent mechanism. FEBS Lett.

[49] Sarkar S (2013). Regulation of autophagy by mTOR-dependent and mTOR-independent pathways: autophagy dysfunction in neurodegenerative diseases and therapeutic application of autophagy enhancers. Biochem Soc Trans.

[50] Leontieva OV, Blagosklonny MV (2016). Gerosuppression by pan-mTOR inhibitors. Aging (Albany NY).

[51] Storer KP, Tu J, Stoodley MA, Smee RI (2010). Expression of endothelial adhesion molecules after radiosurgery in an animal model of arteriovenous malformation. Neurosurgery.

[52] Reddy R, Duong TT, Fairhall JM, Smee RI, Stoodley MA (2014). Durable thrombosis in a rat model of arteriovenous malformation treated with radiosurgery and vascular targeting. J Neurosurg.

[53] McRobb LS, Lee VS, Simonian M, Zhao Z, Thomas SG, Wiedmann M, Raj JV, Grace M, Moutrie V, McKay MJ, Molloy MP, Stoodley MA (2017). Radiosurgery Alters the Endothelial Surface Proteome: Externalized Intracellular Molecules as Potential Vascular Targets in Irradiated Brain Arteriovenous Malformations. Radiat Res.

[54] Maurer K, Torres VJ, Cadwell K (2015). Autophagy is a key tolerance mechanism during Staphylococcus aureus infection. Autophagy.

[55] Postina R, Schroeder A, Dewachter I, Bohl J, Schmitt U, Kojro E, Prinzen C, Endres K, Hiemke C, Blessing M, Flamez P, Dequenne A, Godaux E (2004). A disintegrin-metalloproteinase prevents amyloid plaque formation and hippocampal defects in an Alzheimer disease mouse model. J Clin Invest.

[56] Marples B, McGee M, Callan S, Bowen SE, Thibodeau BJ, Michael DB, Wilson GD, Maddens ME, Fontanesi J, Martinez AA (2016). Cranial irradiation significantly reduces beta amyloid plaques in the brain and improves cognition in a murine model of Alzheimer's Disease (AD). Radiother Oncol.

[57] Pruessmeyer J, Ludwig A (2009). The good, the bad and the ugly substrates for ADAM10 and ADAM17 in brain pathology, inflammation and cancer. Semin Cell Dev Biol.

[58] Reiss K, Saftig P (2009). The “a disintegrin and metalloprotease” (ADAM) family of sheddases: physiological and cellular functions. Semin Cell Dev Biol.

[59] Kuhn PH, Colombo AV, Schusser B, Dreymueller D, Wetzel S, Schepers U, Herber J, Ludwig A, Kremmer E, Montag D, Müller U, Schweizer M, Saftig P (2016). Systematic substrate identification indicates a central role for the metalloprotease ADAM10 in axon targeting and synapse function. eLife.

[60] Maretzky T, Schulte M, Ludwig A, Rose-John S, Blobel C, Hartmann D, Altevogt P, Saftig P, Reiss K (2005). L1 is sequentially processed by two differently activated metalloproteases and presenilin/gamma-secretase and regulates neural cell adhesion, cell migration, and neurite outgrowth. Mol Cell Biol.

[61] Langjahr P, Díaz-Jiménez D, De la Fuente M, Rubio E, Golenbock D, Bronfman FC, Quera R, González MJ, Hermoso MA (2014). Metalloproteinase-dependent TLR2 ectodomain shedding is involved in soluble toll-like receptor 2 (sTLR2) production. PLoS One.

[62] Liu F, Wu S, Ren H, Gu J (2011). Klotho suppresses RIG-I-mediated senescence-associated inflammation. Nat Cell Biol.

[63] Bloch L, Sineshchekova O, Reichenbach D, Reiss K, Saftig P, Kuro-o M, Kaether C (2009). Klotho is a substrate for alpha-, beta- and gamma-secretase. FEBS Lett.

[64] Jin X, Jin X, Jung JE, Beck S, Kim H (2013). Cell surface Nestin is a biomarker for glioma stem cells. Biochem Biophys Res Commun.

[65] Ma J, Sun F, Li C, Zhang Y, Xiao W, Li Z, Pan Q, Zeng H, Xiao G, Yao K, Hong A, An J (2014). Depletion of intermediate filament protein Nestin, a target of microRNA-940, suppresses tumorigenesis by inducing spontaneous DNA damage accumulation in human nasopharyngeal carcinoma. Cell Death Dis.

[66] Muraguchi T, Takegami Y, Ohtsuka T, Kitajima S, Chandana EP, Omura A, Miki T, Takahashi R, Matsumoto N, Ludwig A, Noda M, Takahashi C (2007). RECK modulates Notch signaling during cortical neurogenesis by regulating ADAM10 activity. Nat Neurosci.

[67] Zhao Z, Johnson MS, Chen B, Grace M, Ukath J, Lee VS, McRobb LS, Sedger LM, Stoodley MA (2016). Live-cell imaging to detect phosphatidylserine externalization in brain endothelial cells exposed to ionizing radiation: implications for the treatment of brain arteriovenous malformations. J Neurosurg.

[68] Simonian M, Molloy MP, Stoodley MA (2012). In vitro and in vivo biotinylation of endothelial cell surface proteins in the pursuit of targets for molecular therapies for brain AVMs. Metabolomics.

[69] Krisp C, Yang H, van Soest R, Molloy MP (2015). Online Peptide fractionation using a multiphasic microfluidic liquid chromatography chip improves reproducibility and detection limits for quantitation in discovery and targeted proteomics. Mol Cell Proteomics.

