# Supplementary material for: Ionizing radiation reduces ADAM10 expression in brain microvascular endothelial cells undergoing stress-induced senescence
Source: Aging (Albany NY). 2017 Apr 17;9(4):1248–62. doi: 10.18632/aging.101225 (PMC5425125; doi:10.18632/aging.101225)
Supplement: Supplementary file 1 [file aging-09-1248-s001.pdf]

SUPPLEMENTAL MATERIAL

Table S1. Antibodies used in this study.

Antibody	Catalog Number	Species	Source
P21 (Waf1/Cip1)	sc-6246	Mouse monoclonal	Santa Cruz
MAP L3CB	sc-376404	Mouse monoclonal	Santa Cruz
PAI-1	sc-8979	Rabbit polyclonal	Santa Cruz
ICAM-1	sc-1511R	Rabbit polyclonal	Santa Cruz
TLR2	sc-10739	Rabbit polyclonal	Santa Cruz
NEST	sc-20978	Rabbit polyclonal	Santa Cruz
NEO1	sc-15337	Rabbit polyclonal	Santa Cruz
P16 (CDKN2A)	PAI-46620	Rabbit polyclonal	Thermofisher
α-Tubulin	ab6046	Rabbit polyclonal	Abcam
Caveolin1	ab2910	Rabbit polyclonal	Abcam
Ki67	ab16667	Rabbit monoclonal	Abcam
P62	ab56416	Mouse monoclonal	Abcam
DDX58	ab45428	Rabbit polyclonal	Abcam
ADAM10	ab2124695	Rabbit monoclonal	Abcam
L1CAM	ab24345	Mouse monoclonal	Abcam
GAPDH	ab181602	Rabbit monoclonal	Abcam
Wheat germ agglutinin – AF488	W11261	-	Life Technologies

Table S2. Proteins increased at cell surface (≥ 1.5-fold).

Protein	Fold Change[a]	P value[b]	Protein Name
P22777|PAI1	9.5	0.00	Plasminogen activator inhibitor 1
P97798|NEO1	9.3	0.03	Neogenin
Q9QUN7|TLR2	5.2	0.07	Toll-like receptor 2
P23927|CRYAB	6.8	0.00	Alpha-crystallin B chain
Q9D0J4|ARL2	3.3	0.10	ADP-ribosylation factor-like protein 2
Q6Q899|DDX58	2.9	0.04	Probable ATP-dependent RNA helicase DDX58, retinoic acid inducible gene 1 protein (RIG-1)
P49945|FRIL2	2.8	0.04	Ferritin light chain 2
P07724|ALBU	2.8	0.35	Serum albumin
Q8BHN3|GANAB	2.8	0.10	Neutral alpha-glucosidase AB
Q9DC70|NDUS7	2.8	0.50	NADH dehydrogenase [ubiquinone] iron-sulfur protein 7, mitochondrial
Q8K1N2|PHLB2	2.5	0.11	Pleckstrin homology-like domain family B member 2
O08638|MYH11	2.5	0.05	Myosin-11
Q6PHZ2|KCC2D	2.3	0.43	Calcium/calmodulin-dependent protein kinase type II subunit delta
P11276|FINC	2.3	0.01	Anastellin
Q08879|FBLN1	2.3	0.22	Fibulin-1
Q8BMF4|ODP2	2.2	0.01	Dihydrolipoyllysine-residue acetyltransferase component of pyruvate dehydrogenase complex (PDC-E2), mitochondrial
Q9QZQ1|AFAD	2.1	0.43	Afadin
Q9Z0R6|ITSN2	2.1	0.09	Intersectin-2
Q60680|IKKA	2.0	0.15	Inhibitor of nuclear factor kappa-B kinase subunit alpha
P99029|PRDX5	2.0	0.21	Peroxiredoxin-5, mitochondrial
Q64133|AOFA	1.9	0.00	Amine oxidase [flavin-containing] A
P97770|THUM3	1.9	0.47	THUMP domain-containing protein 3
Q6P5H2|NEST	1.9	0.02	Nestin

P13597|ICAM1	1.9	0.21	Intercellular adhesion molecule 1
P07356|ANXA2	1.8	0.00	Annexin A2
P02089|HBB2	1.8	0.06	Hemoglobin subunit beta-2
Q0KL02|TRIO	1.8	0.00	Triple functional domain protein
Q9WTR1|TRPV2	1.8	0.03	Transient receptor potential cation channel subfamily V member 2
Q9QUM9|PSA6	1.8	0.15	Proteasome subunit alpha type-6
Q64727|VINC	1.8	0.07	Vinculin
P40124|CAP1	1.8	0.00	Adenylyl cyclase-associated protein 1
P85094|ISC2A	1.7	0.02	Isochorismatase domain-containing protein 2A, mitochondrial
Q03963|E2AK2	1.7	0.03	Interferon-induced, double-stranded RNA-activated protein kinase
Q5U430|UBR3	1.7	0.23	E3 ubiquitin-protein ligase UBR3
Q80X90|FLNB	1.7	0.00	Filamin-B
P54116|STOM	1.7	0.01	Erythrocyte band 7 integral membrane protein
Q9CZ30|OLA1	1.7	0.10	Obg-like ATPase 1
Q9Z331|K2C6B	1.7	0.04	Keratin, type II cytoskeletal 6B
O09172|GSH0	1.6	0.28	Glutamate--cysteine ligase regulatory subunit
Q02053|UBA1	1.6	0.05	Ubiquitin-like modifier-activating enzyme 1
P00493|HPRT	1.6	0.01	Hypoxanthine-guanine phosphoribosyltransferase
Q61171|PRDX2	1.6	0.01	Peroxiredoxin-2
Q7TPR4|ACTN1	1.6	0.01	Alpha-actinin-1
P03995|GFAP	1.6	0.14	Glial fibrillary acidic protein
P97434|MPRIP	1.6	0.10	Myosin phosphatase Rho-interacting protein
P26041|MOES	1.6	0.00	Moesin
Q9D6K9|CERS5	1.6	0.21	Ceramide synthase 5
P48678|LMNA	1.6	0.09	Lamin-A/C
Q8BHY3|ANO1	1.6	0.17	Anoctamin-1
Q8C522|ENDD1	1.5	0.16	Endonuclease domain-containing 1 protein
P10107|ANXA1	1.5	0.00	Annexin A1
A6X935|ITIH4	1.5	0.16	Inter alpha-trypsin inhibitor, heavy chain 4
P11627|L1CAM	1.5	0.19	Neural cell adhesion molecule L1
Q9WVL2|STAT2	1.5	0.19	Signal transducer and activator of transcription 2
Q8VED5|K2C79	1.5	0.09	Keratin, type II cytoskeletal 79
P21279|GNAQ	1.5	0.21	Guanine nucleotide-binding protein G(q) subunit alpha
Q80X95|RRAGA	1.5	0.30	Ras-related GTP-binding protein A
Q64261|CDK6	1.5	0.02	Cyclin-dependent kinase 6
Q8CGE9|RGS12	1.5	0.89	Regulator of G-protein signaling 12
P26039|TLN1	1.5	0.25	Talin-1
Q9R112|SQRD	1.5	0.00	Sulfide:quinone oxidoreductase, mitochondrial
Q99PT1|GDIR1	1.5	0.17	Rho GDP-dissociation inhibitor 1
P34884|MIF	1.5	0.01	Macrophage migration inhibitory factor
Q8R2Q8|BST2	1.5	0.20	Bone marrow stromal antigen 2
Q9WUP4|PORED	1.5	0.04	Polyprenol reductase
Q61595|KTN1	1.5	0.05	Kinectin
Q61584|FXR1	1.5	0.35	Fragile X mental retardation syndrome-related protein 1
A2A699|F1712	1.5	0.32	Protein FAM171A2
Q9WTI7|MYO1C	1.5	0.05	Unconventional myosin-Ic
A6H6E2|MMRN2	1.5	0.04	Multimerin-2
Q9EPL8|IPO7	1.5	0.10	Importin-7
P70275|SEM3E	1.5	0.37	Semaphorin-3E
P48036|ANXA5	1.5	0.10	Annexin A5

[a] Fold change (Ratio irradiated/controls; mean of 3 independent experiments). [b] P value (Student's *t*-test).

Table S3. Proteins decreased at the cell surface (≤ 1.5-fold).

Protein	Fold Change[a]	P value[b]	Protein Name
O88342|WDR1	0.2	0.10	WD repeat-containing protein 1
P97310|MCM2	0.2	0.00	DNA replication licensing factor MCM2
P14869|RLA0	0.2	0.02	60S acidic ribosomal protein P0
Q8CGP2|H2B1P	0.3	0.08	Histone H2B type 1-P
P09242|PPBT	0.3	0.00	Alkaline phosphatase, tissue-nonspecific isozyme
P35969|VGFR1	0.3	0.03	Vascular endothelial growth factor receptor 1
P62880|GBB2	0.3	0.01	Guanine nucleotide-binding protein G(I)/G(S)/G(T) subunit beta-2
P16382|IL4RA	0.3	0.02	Interleukin-4 receptor subunit alpha
P08113|ENPL	0.3	0.07	Endoplasmin
Q9D662|SC23B	0.3	0.03	Protein transport protein Sec23B
Q9D7S7|RL22L	0.4	0.02	60S ribosomal protein L22-like 1
Q9CZJ2|HS12B	0.4	0.00	Heat shock 70 kDa protein 12B
P97311|MCM6	0.4	0.06	DNA replication licensing factor MCM6
Q6P4T2|U520	0.4	0.14	U5 small nuclear ribonucleoprotein 200 kDa helicase
Q99JW4|LIMS1	0.4	0.08	LIM and senescent cell antigen-like-containing domain protein 1
Q7TPV4|MBB1A	0.4	0.09	Myb-binding protein 1A
P49718|MCM5	0.4	0.00	DNA replication licensing factor MCM5
Q9JJI8|RL38	0.4	0.01	60S ribosomal protein L38
O35598|ADA10	0.4	0.05	Disintegrin and metalloproteinase domain-containing protein 10
Q9Z2X1|HNRPF	0.4	0.00	Heterogeneous nuclear ribonucleoprotein F, N-terminally processed
P19253|RL13A	0.4	0.01	60S ribosomal protein L13a
Q99J27|ACATN	0.4	0.07	Acetyl-coenzyme A transporter 1
Q62465|VAT1	0.4	0.14	Synaptic vesicle membrane protein VAT-1 homolog
P49717|MCM4	0.4	0.05	DNA replication licensing factor MCM4
Q9QYJ0|DNJA2	0.4	0.04	DnaJ homolog subfamily A member 2
P97857|ATS1	0.4	0.03	A disintegrin and metalloproteinase with thrombospondin motifs 1
Q3TDQ1|STT3B	0.4	0.04	Dolichyl-diphosphooligosaccharide--protein glycosyltransferase subunit
Q91WF3|ADCY4	0.4	0.01	Adenylate cyclase type 4
P13864|DNMT1	0.5	0.02	DNA (cytosine-5)-methyltransferase 1
Q8BK67|RCC2	0.5	0.20	Protein RCC2
P61164|ACTZ	0.5	0.29	Alpha-centractin
P61211|ARL1	0.5	0.10	ADP-ribosylation factor-like protein 1
P62245|RS15A	0.5	0.02	40S ribosomal protein S15a
Q64337|SQSTM	0.5	0.01	Sequestosome-1
P02468|LAMC1	0.5	0.17	Laminin subunit gamma-1
P62918|RL8	0.5	0.67	60S ribosomal protein L8
P15116|CADH2	0.5	0.23	Cadherin-2
Q8R422|CD109	0.5	0.04	CD109 antigen
Q80TN5|ZDH17	0.5	0.02	Palmitoyltransferase ZDHHC17
Q9ES46|PARVB	0.5	0.15	Beta-parvin
Q9CXW4|RL11	0.5	0.02	60S ribosomal protein L11
P62270|RS18	0.5	0.23	40S ribosomal protein S18
Q03145|EPHA2	0.5	0.09	Ephrin type-A receptor 2
P62301|RS13	0.5	0.01	40S ribosomal protein S13
Q6P5D8|SMHD1	0.5	0.27	Structural maintenance of chromosomes flexible hinge domain-containing protein 1
P25206|MCM3	0.5	0.02	DNA replication licensing factor MCM3
P16330|CN37	0.5	0.07	2',3'-cyclic-nucleotide 3'-phosphodiesterase
P97351|RS3A	0.5	0.00	40S ribosomal protein S3a
P11688|ITA5	0.5	0.08	Integrin alpha-5 light chain
O08573|LEG9	0.5	0.05	Galectin-9
Q9JHJ0|TMOD3	0.5	0.00	Tropomodulin-3

Q9D819|IPYR	0.5	0.21	Inorganic pyrophosphatase
Q8BWY3|ERF1	0.5	0.19	Eukaryotic peptide chain release factor subunit 1
Q7TNC4|LC7L2	0.5	0.12	Putative RNA-binding protein Luc7-like 2
Q5SWU9|ACACA	0.5	0.00	Biotin carboxylase
P54761|EPHB4	0.5	0.07	Ephrin type-B receptor 4
P62830|RL23	0.5	0.09	60S ribosomal protein L23
P20029|GRP78	0.5	0.02	78 kDa glucose-regulated protein
P20444|KPCA	0.6	0.11	Protein kinase C alpha type
Q8VDW0|DX39A	0.6	0.04	ATP-dependent RNA helicase DDX39A
Q64151|SEM4C	0.6	0.34	Semaphorin-4C
P02463|CO4A1	0.6	0.79	Arresten
Q9D5V5|CUL5	0.6	0.25	Cullin-5
Q8R1F1|NIBL1	0.6	0.34	Niban-like protein 1
Q01705|NOTC1	0.6	0.30	Neurogenic locus notch homolog protein 1
Q9D8N0|EF1G	0.6	0.10	Elongation factor 1-gamma
P62242|RS8	0.6	0.05	40S ribosomal protein S8
P61620|S61A1	0.6	0.00	Protein transport protein Sec61 subunit alpha isoform 1
Q8BX57|PXK	0.6	0.23	PX domain-containing protein kinase-like protein
Q7M759|AB17B	0.6	0.03	Alpha/beta hydrolase domain-containing protein 17B
P35979|RL12	0.6	0.01	60S ribosomal protein L12
P08775|RPB1	0.6	0.59	DNA-directed RNA polymerase II subunit RPB1
Q9JLV5|CUL3	0.6	0.22	Cullin-3
Q9QUR6|PPCE	0.6	1.00	Prolyl endopeptidase
Q9CU62|SMC1A	0.6	0.89	Structural maintenance of chromosomes protein 1A
Q8R1M2|H2AJ	0.6	0.26	Histone H2A.J
Q60605|MYL6	0.6	0.16	Myosin light polypeptide 6
P11440|CDK1	0.6	0.84	Cyclin-dependent kinase 1
P62908|RS3	0.6	0.01	40S ribosomal protein S3
O55222|ILK	0.6	0.07	Integrin-linked protein kinase
P13439|UMPS	0.6	0.13	Orotidine 5'-phosphate decarboxylase
Q9Z0L0|TPBG	0.6	0.15	Trophoblast glycoprotein
Q05793|PGBM	0.6	1.00	Basement membrane-specific heparan sulfate proteoglycan core protein
P51410|RL9	0.6	0.02	60S ribosomal protein L9
B2RU80|PTPRB	0.6	0.03	Receptor-type tyrosine-protein phosphatase beta
Q80U95|UBE3C	0.6	0.20	Ubiquitin-protein ligase E3C
Q61739|ITA6	0.6	0.09	Integrin alpha-6 light chain
Q9D1R9|RL34	0.6	0.58	60S ribosomal protein L34
O70475|UGDH	0.6	0.99	UDP-glucose 6-dehydrogenase
P14131|RS16	0.6	0.00	40S ribosomal protein S16
Q9JIK5|DDX21	0.6	0.07	Nucleolar RNA helicase 2
P97333|NRP1	0.6	0.11	Neuropilin-1
Q9D0E1|HNRPM	0.6	0.00	Heterogeneous nuclear ribonucleoprotein M
Q8BP67|RL24	0.6	0.01	60S ribosomal protein L24
Q9D198|SYF2	0.6	0.89	Functional Spliceosome-Associated Protein 29
P17225|PTBP1	0.6	0.26	Polypyrimidine tract-binding protein 1
Q9JJ28|FLII	0.6	0.12	Protein flightless-1 homolog
O35286|DHX15	0.6	0.31	Putative pre-mRNA-splicing factor ATP-dependent RNA helicase
P70168|IMB1	0.6	0.09	Importin subunit beta-1
O35218|CPSF2	0.6	0.25	Cleavage and polyadenylation specificity factor subunit 2
Q9D8Z1|ASCC1	0.6	0.99	Activating signal cointegrator 1 complex subunit 1
Q9QZM4|TR10B	0.6	0.03	Tumor necrosis factor receptor superfamily member 10B
Q8BIJ6|SYIM	0.6	0.32	Isoleucine--tRNA ligase, mitochondrial
P16056|MET	0.6	0.06	Hepatocyte growth factor receptor
P97363|SPTC2	0.6	0.34	Serine palmitoyltransferase 2
Q62167|DDX3X	0.6	0.23	ATP-dependent RNA helicase DDX3X

Q8BTJ4|ENPP4	0.6	0.99	Bis(5'-adenosyl)-triphosphatase enpp4
Q9JL15|LEG8	0.6	0.04	Galectin-8
P61358|RL27	0.6	0.01	60S ribosomal protein L27
Q3UH93|PLXD1	0.6	0.19	Plexin-D1
Q02257|PLAK	0.6	0.05	Junction plakoglobin
O70503|DHB12	0.6	0.35	Very-long-chain 3-oxoacyl-CoA reductase
Q05920|PYC	0.6	0.21	Pyruvate carboxylase, mitochondrial
P62849|RS24	0.6	0.10	40S ribosomal protein S24
P80315|TCPD	0.6	0.00	T-complex protein 1 subunit delta
P12382|K6PL	0.6	0.99	ATP-dependent 6-phosphofructokinase, liver type
P35293|RAB18	0.6	0.32	Ras-related protein Rab-18
Q9D8E6|RL4	0.6	0.01	60S ribosomal protein L4
O89103|C1QR1	0.6	0.06	Complement component C1q receptor
O54890|ITB3	0.6	0.85	Integrin beta-3
P23116|EIF3A	0.6	0.99	Eukaryotic translation initiation factor 3 subunit A
B2RXS4|PLXB2	0.6	0.16	Plexin-B2
O88746|TOM1	0.6	0.34	Target of Myb protein 1
Q61024|ASNS	0.6	0.01	Asparagine synthetase [glutamine-hydrolyzing]
P63037|DNJA1	0.6	0.21	DnaJ homolog subfamily A member 1
Q62351|TFR1	0.7	0.09	Transferrin receptor protein 1
Q60865|CAPR1	0.7	0.13	Caprin-1
Q99LC5|ETFA	0.7	0.34	Electron transfer flavoprotein subunit alpha, mitochondrial
Q06806|TIE1	0.7	0.29	Tyrosine-protein kinase receptor Tie-1
P16110|LEG3	0.7	0.11	Galectin-3
P55284|CADH5	0.7	0.19	Cadherin-5
Q9CZR2|NALD2	0.7	0.49	N-acetylated-alpha-linked acidic dipeptidase 2
Q9DCD0|6PGD	0.7	0.28	6-phosphogluconate dehydrogenase, decarboxylating

[a] Fold change (Ratio irradiated/controls; mean of 3 independent experiments). [b] P value (Student's *t*-test).

Table S4. Ingenuity pathway analysis.

**TOP CANONICAL PATHWAYS**		
**Name**	**p-value**	**Overlap**
EIF2 signalling	2.47E-34	27.6% 51/185
Regulation of eIF4 and p70S6K signalling	1.01E-17	21.2% 31/146
Epithelial adherens junction signalling	6.12E-15	19.2% 28/146
Caveolar-mediated endocytosis signalling	2.56E-13	26.8% 19/71
Germ cell-sertoli cell junction signalling	4.87E-13	16.9% 27/160
**TOP NETWORKS**		
**Name**	**p-value**	**# molecules**
Cellular movement	1.79E-05 – 4.51E-32	214
Cellular growth and proliferation	8.08E-06 – 5.18E-29	285
Cellular assembly and organization	8.13E-06 – 3.12E-26	211
Cellular function and maintenance	1.79E-05 – 3.12E-26	249
Protein synthesis	1.26E-05 – 2.77E-24	139
**TOP TOX LISTS**		
**Name**	**p-value**	**Overlap**
Renal necrosis/cell death	3.00E-09	8.5% 42/496
NRF2-mediated oxidative stress response	3.73E-05	8.5% 20/234
PPAR/RXR activation	4.99E-05	9.3% 17/183
Hypoxia-inducible factor signalling	2.74E-04	12.9% 9/70
Mitochondrial dysfunction	3.50E-04	8.5% 15/176
**TOP UPSTREAM REGULATORS**		
**Upstream Regulator**	**p-value of overlap**	**Predicted Activation**
MYC	1.28E-40	Inhibited
MYCN	1.37E-37	Inhibited
TP53	1.80E-34	-
sirolimus	4.69E-31	Activated
5-fluorouracil	7.19E-28	Activated
